# Sevoflurane promotes premature differentiation of dopaminergic neurons in hiPSC-derived midbrain organoids

**DOI:** 10.3389/fcell.2022.941984

**Published:** 2022-09-13

**Authors:** Jia Shang, Bin Li, Han Fan, Peidi Liu, Wen Zhao, Tao Chen, Pu Chen, Longqiu Yang

**Affiliations:** ^1^ Tissue Engineering and Organ Manufacturing (TEOM) Lab, Department of Biomedical Engineering, TaiKang Medical School (School of Basic Medical Sciences), Wuhan University, Wuhan, Hubei, China; ^2^ Department of Anesthesiology, Huangshi Central Hospital, Affiliated Hospital of Hubei Polytechnic University, Huangshi, Hubei, China; ^3^ Hubei Province Key Laboratory of Allergy and Immunology, Wuhan, Hubei, China; ^4^ Medical College, Wuhan University of Science and Technology, Wuhan, Hubei, China; ^5^ TaiKang Medical School (School of Basic Medical Sciences), Wuhan University, Wuhan, Hubei, China

**Keywords:** sevoflurane, midbrain organoids, dopaminergic neurons, differentiation, premature, fetal brain development

## Abstract

**Background:** Conventional animal models used in corresponding basic studies are distinct from humans in terms of the brain’s development trajectory, tissue cytoarchitecture and cell types, making it difficult to accurately evaluate the potential adverse effects of anesthetic treatments on human fetal brain development. This study investigated the effects of sevoflurane on the midbrain’s development and cytopathology using human physiologically-relevant midbrain organoids.

**Methods:** Monolayer human induced pluripotent stem cells (hiPSC)-derived human floor plate cells and three-dimensional hiPSC-derived midbrain organoids (hMBOs) were exposed to 2% (v/v) sevoflurane for 2 or 6 h, followed by expansion or differentiation culture. Then, immunofluorescence, real-time PCR, EdU assay, Tunnel assay, and transcriptome sequencing were performed to examine the effects of sevoflurane on the midbrain’s development.

**Results:** We found that 2% sevoflurane exposure inhibited hFPCs’ proliferation (differentiation culture: 7.2% ± 0.3% VS. 13.3% ± 0.7%, *p* = 0.0043; expansion culture: 48% ± 2.2% VS. 35.2% ± 1.4%, *p* = 0.0002) and increased their apoptosis, but did not affect their differentiation into human dopaminergic neurons After 6 h, 2% sevoflurane exposure inhibited cell proliferation (62.8% ± 5.6% VS. 100% ± 5.5%, *p* = 0.0065) and enhanced the premature differentiation of hMBOs (246% ± 5.2% VS. 100% ± 28%, *p* = 0.0065). The RNA-seq results showed long-term exposure to sevoflurane up regulates some transcription factors in the differentiation of dopaminergic neurons, while short-term exposure to sevoflurane has a weak up-regulation effect on these transcription factors.

**Conclusion:** This study revealed that long-term exposure to sevoflurane could promote the premature differentiation of hMBOs, while short-term exposure had negligible effects, suggesting that long-term exposure to sevoflurane in pregnant women may lead to fetals’ midbrain development disorder.

## Introduction

Sevoflurane is one of the most commonly used inhaled anesthetic agents during the embryonic period or before 3 years old due to its low blood-gas partition coefficient, fast onset of action, rapid elimination, and high hemodynamic stability (De Hert and Moerman, 2015; Palanca et al., 2017). Recent animal studies indicated that sevoflurane treatment at the clinical dose could induce multiscale neuropathological features in fetal mice, including cognitive impairment, neuronal apoptosis, synaptic deficiency, and neuroinflammation (Amrock et al., 2015; Makaryus et al., 2015; Ozer et al., 2017; Yu et al., 2020; Dai et al., 2021). A recent prospective study revealed that children with multiple exposures to anesthetic surgeries had a higher probability of developing fine motor impairment, despite not displaying significant reductions in intelligence quotient (Warner et al., 2018). In 2016, the U.S. FDA warned that repetitive or long-term use of general anesthetic agents during surgeries or procedures in children younger than 3 years or pregnant women during their third trimester might impair the children’s/fetuses’ neurodevelopment, but the underlying mechanisms remain poorly understood.

Dopaminergic neurons (DANs), a neuronal subtype enabling the synthesis and release of dopamine as a neurotransmitter, widely exist in various brain regions in mammals (Hegarty et al., 2013). Specifically, DANs are mainly located in the midbrain and regulate the extrapyramidal system’s motor functions (Warner et al., 2018). Recent animal studies indicated that DANs played a critical role in modulating the emergence of rats from sevoflurane anesthesia (Gui et al., 2021). Moreover, sevoflurane exposure was shown to significantly increase dopamine release in rats’ brain (Toner et al., 2001; Silva et al., 2007). However, the effects of sevoflurane on human DAN (hDAN) differentiation during human fetal brain development remain unclear (Li et al., 2019) due to the lack of human physiologically-relevant research models.

Human brain organoid (hBO) is an emerging human-induced pluripotent stem cell (hiPSCs)-derived brain research model that can closely emulate some critical neurophysiological features of the human brain, including neurodevelopmental trajectory, neural cell diversity, and cytoarchitectures (Lancaster et al., 2013; Velasco et al., 2019; Jacob et al., 2020; Qiao et al., 2020). In particular, hiPSCs-derived midbrain organoid (hMBO) is a specific type of hBO that contains an hDAN-rich cell population. Previous transcriptome profile analyses indicated that hMBOs resembled human prenatal midbrain more closely than monolayer hDANs (Fasano et al., 2010; Jo et al., 2016; Lin et al., 2016). Additionally, confocal immunofluorescence and neuro-electrophysiological analysis demonstrated that hDANs formed complex functional neurosynaptic networks with spontaneous neuronal activity in hMBOs (Monzel et al., 2017). Thus, hMBOs have been widely used in mechanistic and drug discovery studies of hDAN-related brain diseases, such as Parkinson’s disease (Galet et al., 2020; Marotta et al., 2020; Smits and Schwamborn, 2020; Capucciati et al., 2021; Fiorenzano et al., 2021).

In this study, we generated monolayer hFPCs and three-dimensional hMBOs from hiPSCs as research models for human midbrain development to examine sevoflurane exposure-induced multiscale neuropathology from the aspects of proliferation, differentiation and apoptosis.

## Materials and methods

### Generation of hMBOs

hMBOs were constructed using a previously reported protocol (Monzel et al., 2017). Human neuroepithelial stem cells (hNSCs) were cultured in an N2B27 maintenance medium (Supplementary Table S1). On day 0 (D0), 10,000 hNSCs were seeded into each well of ultra-low adhesion 96-well U-shaped plate (Corning Company, 7007) and cultured in an N2B27 maintenance medium (Supplementary Table S2). The medium was changed every other day. On D8, the organoids were coated with Matrigel and transferred to an ultra-low adhesion 24-well plate (Corning Company, 3473), with one organoid in each well. On D10, differentiation was induced using the N2B27 differentiation medium (Supplementary Table S3). On D14, the 24-well plate was transferred to an orbital shaker rotating at 80 rpm in an incubator (5% CO2, 37°C), and the medium was changed every other day. On D16, differentiation was continued using the N2B27 differentiation medium without purmorphamine.

### Differentiation of monolayer hDANs from hiPSCs

hiPSCs were cultured in mTeSR (STEMCELL Technologies, 85850) with 5 μm ROCK inhibitor (Y27632) medium (STEMCELL Technologies, 72304). The differentiation of dopaminergic neurons using a PSC Dopaminergic Neuron Differentiation Kit (Thermo Fisher, A30416SA), following the manufacturer’s instructions. First, hiPSCs were seeded in a 6-well plate at a confluence of 25% and cultured in a mTeSR medium. On D1, the cells were induced in FP cells differentiation medium in FP Specification Medium. On D10, the FP cells reached 100% confluence and were passaged for 2 to 5 generations in complete FP Cell expansion medium. Finally, the FP cells were seeded with Dopaminergic Neuron Maturation Medium containing 5 μM ROCK inhibitor at a density of 1.0 × 10^5^ cells cm^−2^ on a 6- or 48-well plate coated with poly-lysine and laminin. Mature neurons were characterized after 14 days of culture.

### Sevoflurane exposure

Sevoflurane exposure was performed as illustrated in Supplementary Figure S1. It was composed of 5 parts: a volatile sevoflurane tank, a pump, an exposure box, a sevoflurane sensor, and a waste gas recovery device. The Sevoflurane exposure box was placed in a cell incubator. The gas (CO_2_:5%, temperature: 37°C, humidity:95%) was pumped from the cell incubator to the volatile sevoflurane tank. Then, gas with 2% sevoflurane was pumped to the exposure box at a flow rate of 1 L min^−1^, the control group was treated with the gas (CO_2_:5%, temperature: 37°C, humidity:95%) with 0% sevoflurane at a flow rate of 1 L min-1, while the treatment group is the 2% sevoflurane in the gas (CO2:5%, temperature: 37°C, humidity:95%). The redundant gas was recycled by the waste gas recovery device. The sevoflurane concentration in the exposure box was determined by the sevoflurane sensor.

### Characterization of the organoids’ growth curve

To characterize the organoid growth curve, the bright-field images of hMBOs were acquired on days 1, 6, 11, 16 and 21 via an Olympus microscope IX83. Image analysis was conducted using the ImageJ software (National Institutes of Health, Bethesda, MD, USA) to determine the average diameter of the organoids.

### Lactate dehydrogenase assay

On D1, hFPCs were seeded into 96-well plates at a confluence of 30%. The culture wells were divided into the following five groups: (De Hert and Moerman, 2015): cell-free medium wells (background blank control), (Palanca et al., 2017), untreated wells with subsequent cell lysis (sample maximum enzyme activity), (Amrock et al., 2015), untreated wells (drug-untreated sample control), and treated wells ([4] short and [5] long-term sevoflurane treated samples). On D2, the cells were exposed to 2% (v/v) sevoflurane for 2 or 6 h, followed by subsequent normal culture for 48 h. The cell supernatant in the varied groups was collected for LDH release detection.

LDH release detection was conducted using an LDH detection kit (Beyotime, C0016) following the manufacturer’s protocol (www.beyotime.com/product/C0016.htm). Specifically, the absorbance was then measured at 490 nm using a multifunctional plate reader (INFINITE 200 PRO). LDH release was calculated using the following Eq. 1:

$$\begin{array}{l} \text{LDH release percentage}\\ =\frac{\text{measured absorbance of each group }‐\text{ absorbance of background blank control well}}{\text{absorbance of maximum enzyme activity of cells}‐\text{ absorbance of background blank control well}}\times 100\% \end{array}$$ (1)

### EdU labeling assay

On D1, hFPCs were seeded into 96-well plates at a confluence of 30%. On D2, the cells were exposed to 2% (v/v) sevoflurane for 2 or 6 h. The treated cells were immersed with an expansion culture or differentiation culture for 72 h. We explored different EdU incubation times (10 min, 1 h, 4 h) according to the proliferation rate of FPCs before the experiment, and finally determined to incubate for 1 h. EdU (Beyotime, C0071) was added to the cell culture plate to form a final working concentration of 10 µM. The cells were incubated with the EdU at 37°C for 1 h. Fluorescence images of EDU and DAPI stained cells were taken using an Olympus fluorescence microscope. Fluorescence images of EDU and DAPI stained cells were taken using an Olympus fluorescence microscope. EdU positive cell rate was calculated using the following Eq. 2.

$$\text{EdU positive cell rate}=\frac{\text{Number of EdU positive cells}}{\text{Number of DAPI}}\times 100\%$$ (2)

### TUNEL assay

The sample and control groups for TUNEL assays were the same as the EDU assay. TUNEL assay was conducted using a TUNEL detection kit (Beyotime, C1086) following the manufacturer’s protocol (www.beyotime.com/product/C1086.htm). Fluorescence images of TUNEL and DAPI stained cells were acquired with an Olympus fluorescence microscope. The excitation wavelength range was 450–500 nm, and the emission wavelength range was 515–565 nm (green fluorescence). TUNEL positive cell rate was calculated using the following Eq. 3.

$$\text{TUNEL positive cell rate }=\frac{\text{Number of TUNEL positive cells}}{\text{Number of DAPI}}\times 100\%$$ (3)

### Immunofluorescence

The cells were fixed with 4% (w/v) paraformaldehyde for 15 min. The cells were sealed in the PBS with 0.3% (v/v) Triton X-100 and 3% (v/v) normal donkey serum for 1 h. The cells were then incubated with the primary antibody solution overnight at 4°C. After washing three times with PBS, the cells were incubated with a secondary antibody. The nucleus was stained with DAPI (Life Technologies). The immunofluorescence images were taken with an Olympus X83 microscope. Image processing and analysis were performed using the ImageJ software (NIH, MD, USA).

For immunofluorescence, the midbrain organoids were fixed in 4% (w/v) paraformaldehyde at 4°C overnight. Sections of the sample were 8 μm in thickness (Leica CM 1950). Images were taken with a Leica TCS SP8 STED confocal microscope equipped with LAS X software. To quantify protein distribution, all micrographs were taken using the same laser intensity detector for sensitivity, amplification and offset. The detailed information on the antibodies are shown in Supplementary Table S4. Quantitative analysis of immunofluorescence images was conducted using the ImageJ software using the following Eq. 4, 5.

Eq. 4:

$$\text{Positive rate of nuclear protein }=\frac{\text{Number of target protein}-\text{Positive cells}}{\text{Number of DAPI}}\times 100\%$$ (4)

Eq. 5:

$$\text{Fluorescence intensity of cytoplasm protein }=\frac{\text{Fluorescence intensity of target protein}}{\text{DAPI area}}\times 100\%$$ (5)

### Real-time PCR

Total RNA was isolated from hMBOs and hFPCs using a Trizol reagent. cDNA was synthesized using AccuPower RT-PCR PreMix (Bioneer). Real-time PCR was performed using SYBR Green Real-Time PCR Master Mixes (Invitrogen) under 40 cycles with the following conditions: denaturation at 95°C for 1 min, annealing at 58°C for 30 s, and extension at 72°C for 30 s. The primer sequences are shown in Supplementary Table S5. The expression level of the housekeeping gene GAPDH was normalized using the comparative Ct method: 2^−ΔΔCt.^

### Transcriptome sequencing

hMBOs were exposed to sevoflurane on D8 and divided into the following three groups: (De Hert and Moerman, 2015): MBO-SF-2%2H group (exposure to 2% sevoflurane for 2 h), (Palanca et al., 2017), MBO-SF-2%6H group (exposure to 2% sevoflurane for 6 h), and (Amrock et al., 2015) control group (no exposure to sevoflurane). After the exposure, the incubation medium was replaced with an N2B27 differentiation medium for subsequent differentiation culture until D16 for RNA extraction. RNA quality control was performed to check the purity, concentration and integrity of the RNA. After passing the test, RNA sequencing was performed using Illumina Next Seq6000, with an average of 20 million reads per run. The DAVID database was used for gene ontology (GO) enrichment analysis. To obtain the astringent DEG data set, only DEGs with ≥1.5-fold change was used for GO analysis. mRNA-seq technical support and data analysis was obtained from Seqhealth Technology Company Limited. The transcriptomics data have been submitted to the Sequence Read Archive (database identifier: PRJNA825742). We downloaded the transcriptome data of two-dimensional dopaminergic neurons from hiPSC (GIS_2D_DAN), midbrain organoids from hiPSC (GIS_hMLO) and the human prenatal midbrain (GIS_Midbrain) from the public database (Jo et al., 2016) and compared them with hMBOs.

### Data statistical methods

All data were analyzed using GraphPad Prism version 6.0. The Shapiro-Wilk test, D'Agostino-Pearson test, or Kolmogorov-Smirnov test was used for normal distribution analysis. The statistical significance was determined by One-way ANOVA or SNK-q test when variables followed normal distribution. Variables following skewed distribution statistical significance were determined by the Kruskal–Wallis test or Dunn’s multiple comparison test. The data presented as mean ± SEM. A *p*-value of <0.05 was considered to indicate significant differences. In all analyses, group differences were considered statistically significant, as shown below: *p ≤ 0.05, **p ≤ 0.01, ***p ≤ 0.005, ****p ≤ 0.001. n. s was no statistical difference.

## Results

### Sevoflurane inhibited the proliferation of monolayer hFPCs

**FIGURE 1:**
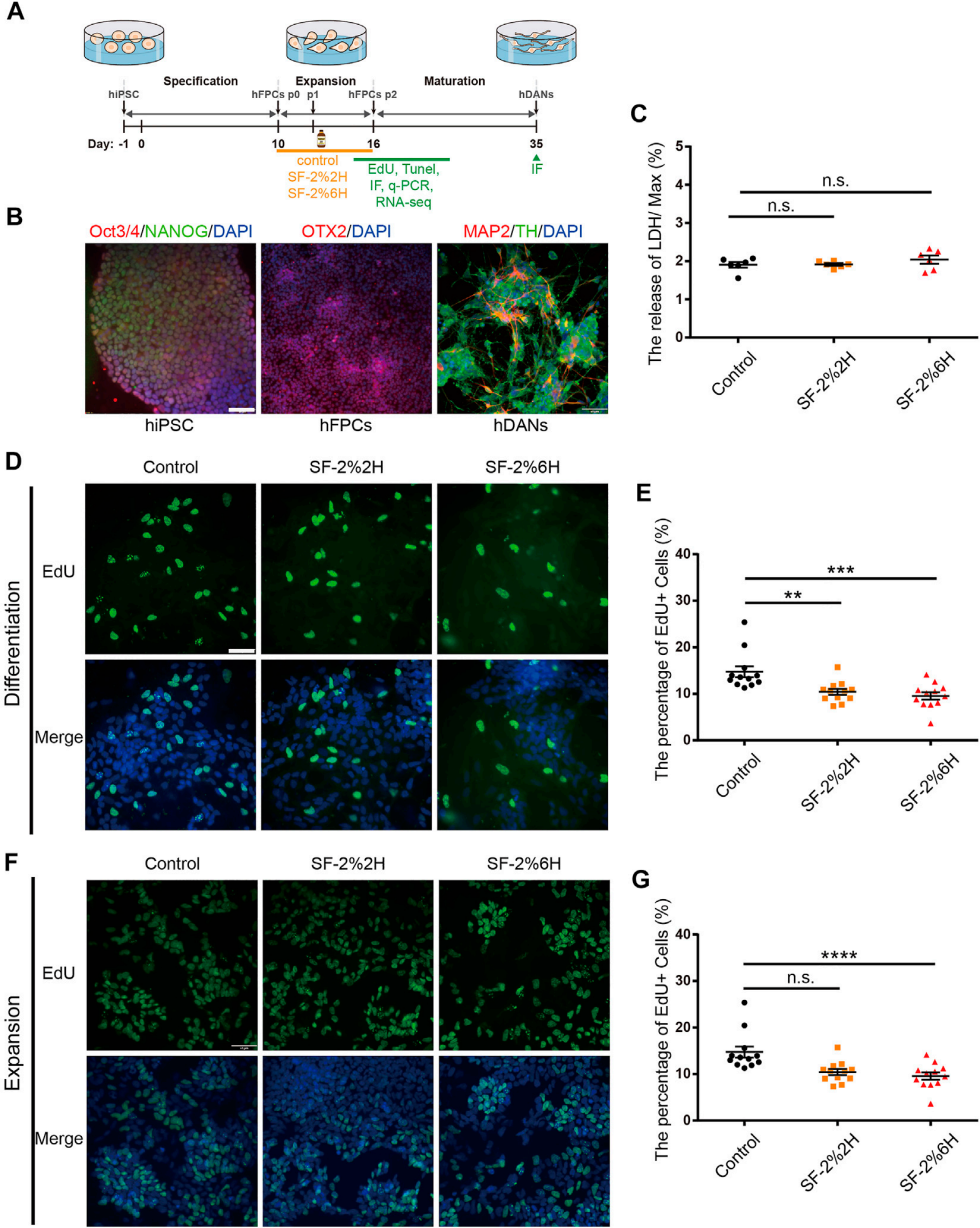
Sevoflurane decreased the proliferation of monolayer hFPCs. **(A)** Schematic representation of the experimental pipeline used in the differentiation process of hiPSCs-derived hDANs. **(B)** Confirmation of the expressions of hiPSCs, hiPSCs-derived hFPCs, and hFPCs-derived hDANs. Immunolabeling of Oct4 (red), Nanog (green) and DAPI (blue) in hiPSCs; OTX2 (red) in hiPSCs-derived hFPCs on D10. hFPCs were further differentiated into hDANs, as illustrated using MAP2 (red) and TH (green) on D35. **(C)** LDH relative release of hFPCs in the control, SF-2%2H and SF-2%6H groups. **(D–G)** Representative image of EdU staining (green) with DAPI (blue) counterstain during both differentiation **(D)** and expansion **(F)**. **(E)** Quantification of **(D)**. **(G)** Quantification of **(F)**. Sample images of hFPCs were taken using an Olympus microscope IX83 **(D,F)**. Scale bars represent 50 μm. Data represent mean ± SEM (*n* = 12).

The differentiation of hiPSCs into hFPCs and hDANs was performed using the protocol illustrated in Figure 1A. On D0, immunofluorescence analysis indicated that nearly 100% of hiPSCs expressed OCT3/4 and NANOG (PSC-specific markers), revealing their pluripotent identity. On D10, more than 90% of the differentiated cells expressed Orthodenticle Homeobox 2 (OTX2, hFPCs-specific marker), revealing their hDAN progenitor’s destiny. On D35, more than 30% of the differentiated cells co-expressed microtubule-associated protein 2 (MAP2, post-mitotic mature neuron-specific marker) and Tyrosine hydroxylase (TH, hDAN-specific marker), revealing their matured hDAN identity (Figure 1B).

Monolayer hFPCs were exposed to 2% sevoflurane for 2 h (SF-2%2H group, short-term exposure) and 6 h (SF-2%6H group, long-term exposure), and another set of monolayer hFPCs was not exposed to sevoflurane (control group). Their corresponding level of plasma membrane damage on the hFPCs were then assessed. The results showed no significant difference in the amount of LDH released from hFPCs in both the SF-2%2H group and SF-2%6H group compared with the control group (Figure 1C), indicating that sevoflurane may have no significant cytotoxicity to hFPCs.

Next, we assessed the effects of sevoflurane exposure on hFPCs proliferation using the EdU labeling assay in hFPC differentiation and expansion culture conditions. In the differentiation culture condition, the EdU labeling assay showed that the fraction of EdU-positive cells was 10.5% ± 0.5% in the SF-2%2H group and 7.2% ± 0.3% in the SF-2%6H group, while the fraction of EdU-positive cells was 13.3% ± 0.7% in the control group, indicating that sevoflurane inhibited hFPC proliferation in the differentiation culture (Figures 1D,E). In the expansion culture condition, the fraction of EdU-positive cells in the SF-2%2H group (47.3% ± 2.7%) showed no significant difference compared with the control group (48% ± 2.2%). Conversely, the fraction of EdU-positive cells in the SF-2%6H group (35.2% ± 1.4%) was significantly reduced compared with the control group (Figures 1F,G), indicating that long-term sevoflurane exposure inhibited hFPCs proliferation in the expansion culture.

### Sevoflurane increased apoptosis of hFPCs

**FIGURE 2:**
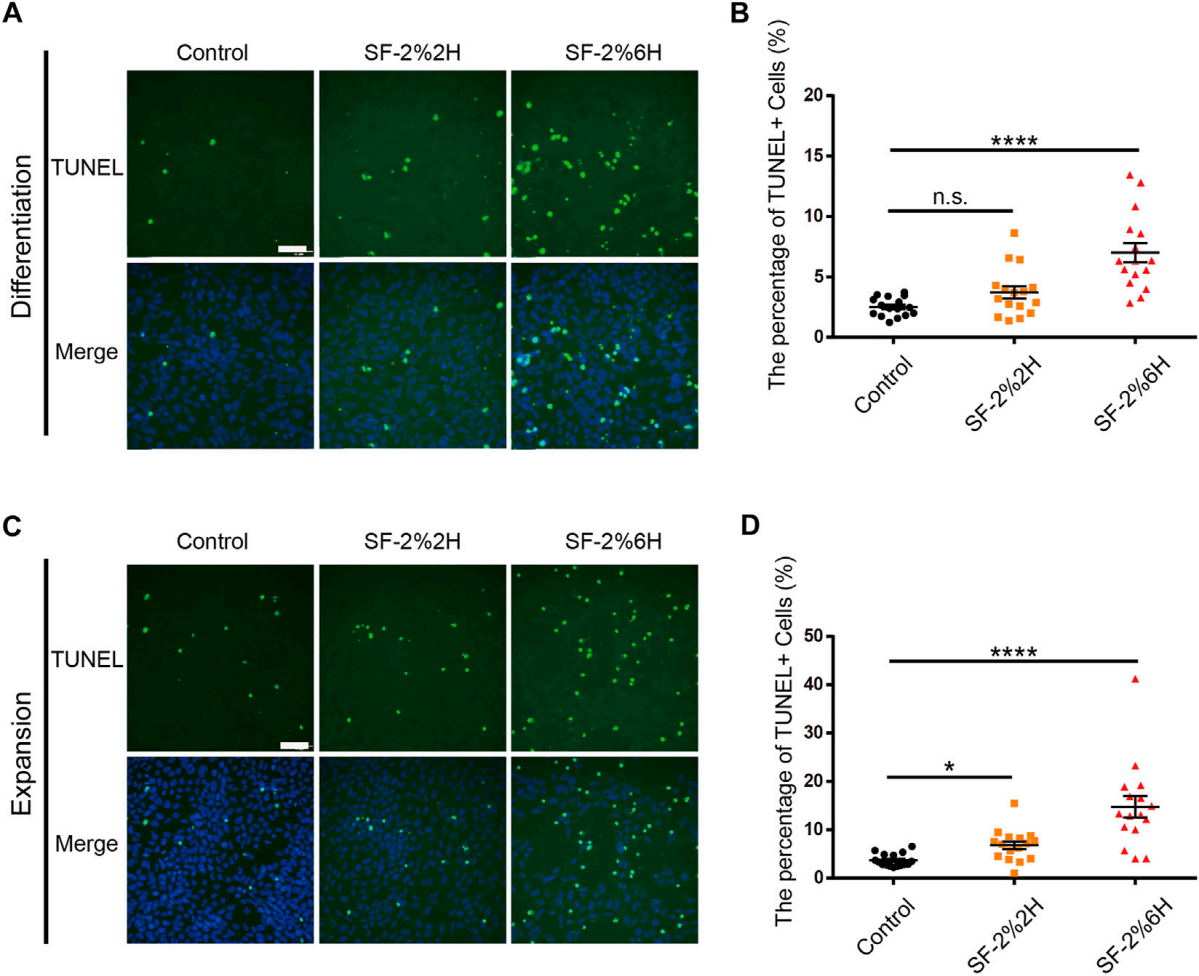
Sevoflurane increased the apoptosis of monolayer hFPCs. **(A–D)** Representative image of TUNEL staining (green) with DAPI (blue) counterstain during both differentiation **(A)** and expansion **(C)**. **(B)** Quantification of **(A)**. **(D)** Quantification of **(C)**. Sample images of hFPCs were taken using an Olympus microscope IX83. Data represent mean ± SEM (*n* = 16).

Here, we determined the effects of sevoflurane exposure on hFPC apoptosis using TUNEL assays in both hFPC differentiation and expansion culture conditions. In the differentiation culture condition, the results showed that the fraction of TUNEL-positive cells was 7.0% in the SF-2%2H group and 14.1% in the SF-2%6H group compared with 3.8% in the control group (Figures 2A,B). In the expansion culture condition, the fraction of TUNEL-positive cells was 2.0% in the SF-2%2H group and 3.8% in the SF-2%6H group compared with 1.2% in the control group. These results indicated that sevoflurane exposure promoted hFPC apoptosis, regardless of the culture conditions (Figures 2C,D).

### Sevoflurane showed no significant effect on the monolayer differentiation of hFPCs into hDANs

In this section, we investigated the effects of sevoflurane on monolayer differentiation of hFPCs into hDANs using immunofluorescence analysis. Immunofluorescence for the intermediate filament Nestin (NES) and transcription factor SOX-2 (SOX2) was used to discriminate CNS stem and progenitor cells from more differentiated progeny. Immunofluorescence for the tubulin beta-3 (TUBB3) was used to identify early neurons, and that for tyrosine hydroxylase (TH) to differentiate hDANs from other neurons.

Immunofluorescence quantitative analysis demonstrated that the protein expression levels of NES (Supplementary Figures S2A, B), SOX2 (Supplementary Figures S2A,C), TUBB3 (Supplementary Figures S3A,B) and TH (Supplementary Figure S3D,E) either in the SF-2%2H group or the SF-2%6H group was not significantly different from the control group. The results of their mRNA level were consistent with their protein level results (Supplementary Figure S2D,E, Supplementary Figure S3C,F), indicating that sevoflurane had a negligible effect on the monolayer differentiation of hFPCs into hDANs.

### hMBOs mimic the characteristics of the early development of the human midbrain

**FIGURE 3:**
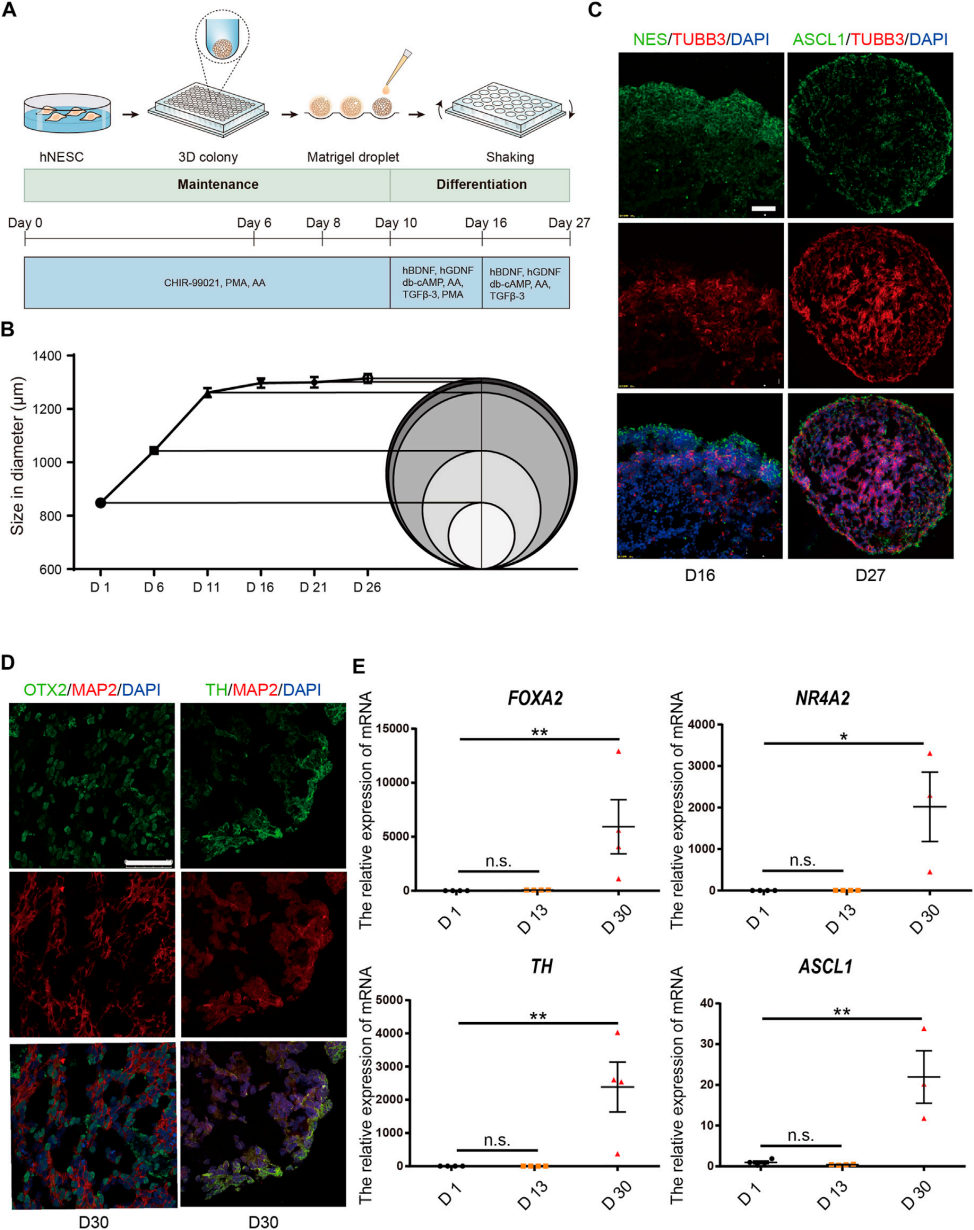
hMBOs mimicking the early stages of human midbrain development. **(A)** Schematic diagram of the hMBOs method and timing. **(B)** Diameter changes of hMBOs. **(C)** The immunofluorescence staining for NES (green), TUBB3 (red) on D16, and ASCL1 (green), TUBB3 (red) and DAPI (blue) on D27. **(D)** The immunofluorescence staining for OTX2 (green), MAP2 (red), TH (green) and DAPI (blue) in hMBOs on D27. **(E)** Detection of the mRNA expression of FOXA2, NURR1, TH and ASCL1. Images were captured on a Leica TCS SP8 STED confocal microscope. Scale bars: 50 μm.

The generation of hMBOs from hiPSC is illustrated in Figure 3A. We found that hMBOs rapidly increased in size in the first 11 days and reached a mean core size of 1.3 ± 0.02 mm in diameter on D26 (Figure 3B). To determine the maturity of the hMBOs, corresponding protein markers of hMBOs were assessed on D16 and D27. Our results showed that TUBB3-positive cells began to appear in hMBOs on D16; at the same time, a large number of NES-positive cells was observed in hMBOs, indicating that only a small part of neural stem cells had differentiated into early-stage neurons (Figure 3C). We also found that TUBB3-positive cells were significantly increased from D16 to D27. Mature neuron marker microtubule-associated protein 2 (MAP2) was also expressed on D30 and could be co-expressed with OTX2 or TH (Figure 3D), suggesting that mature hDANs were produced in hMBOs on D30. These data indicate that cells in the hMBOs gradually transitioned from proliferating neuron progenitors to post-mitotic mature neurons.

**FIGURE 4:**
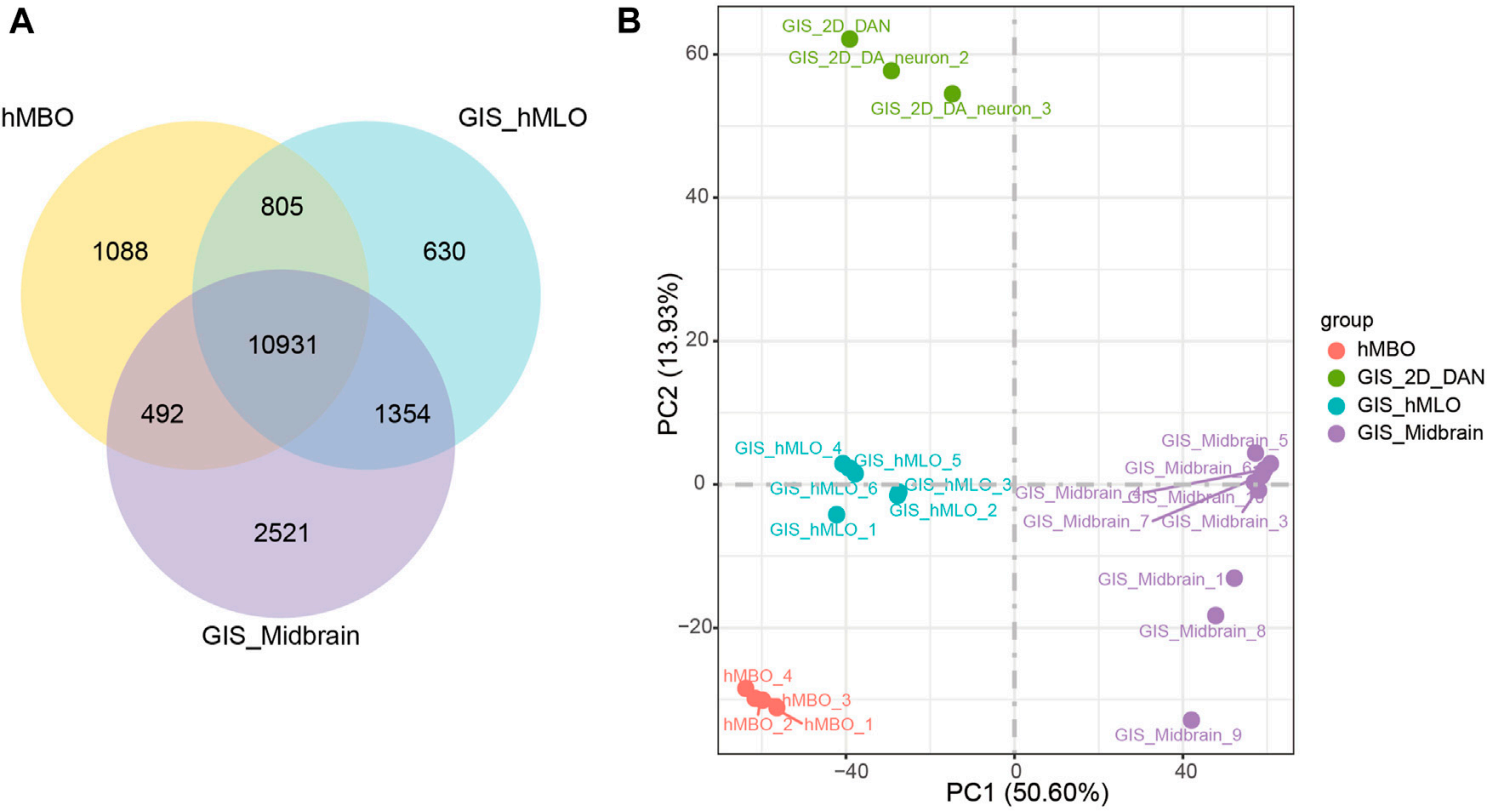
hMBOs mimicking the human midbrain in transcription. **(A)** DGE analysis among the hMBO, GIS_hMLO and GIS_Midbrain group. **(B)** PCA shows a clear separation between GIS_2D_DAN and the other groups.

The mRNA levels of forkhead box A2 (FOXA2), nuclear receptor subfamily four group A member 2 (NR4A2) and achaete-scute family bHLH transcription factor 1 (ASCL1) are important transcription factors in the differentiation process of hDANs, and were also found to be increased with the differentiation line of hMBOs (Figure 3E). The transcriptome expressed in hMBO, GIS_hMLO and GIS_Midbrain were identified on the basis of the human transcriptome atlas. GIS_hMLO showed a high overlap of gene expression with GIS_Midbrain (73%), and the hMBO showed a similar high overlap of gene expression with GIS_Midbrain (66%). (Figure 4A). Then, the principal component analysis (PCA) was calculated using all genes in normalized read counts across all samples (regularized log transformation). We analysed the RNA-Seq data from hMBO, GIS_hMLO and GIS_Midbrain and GIS_2D_DAN, the results showed that a comparison with publicly available 2D-DA neuron expression data, the data from midbrain organoid, the human prenatal midbrain and our midbrain organoid data further suggests that GIS_hMLO are closer to the prenatal midbrain, the hMBO showed a similar distance to GIS Midbrain as GIS_hMLO. (Figure 4B). These results may be due to the fact that in order to study the effect of sevoflurane on midbrain development, we selected early differentiated midbrain organoids (D16) for transcriptome sequencing, while the GIS_hMLO were more mature organoids (D45). This explains why the overlapping gene is more than hMBO compared to GIS_Midbrain, and why the distance between hMBO and GIS_Midbrain is far more than the distance between GIS_hMLO and GIS_Midbrain. Taken together, these results indicate hMBOs mimic the transcriptional of the human prenatal midbrain.

### Sevoflurane promoted the early development of hMBOs at the transcriptional level

**FIGURE 5:**
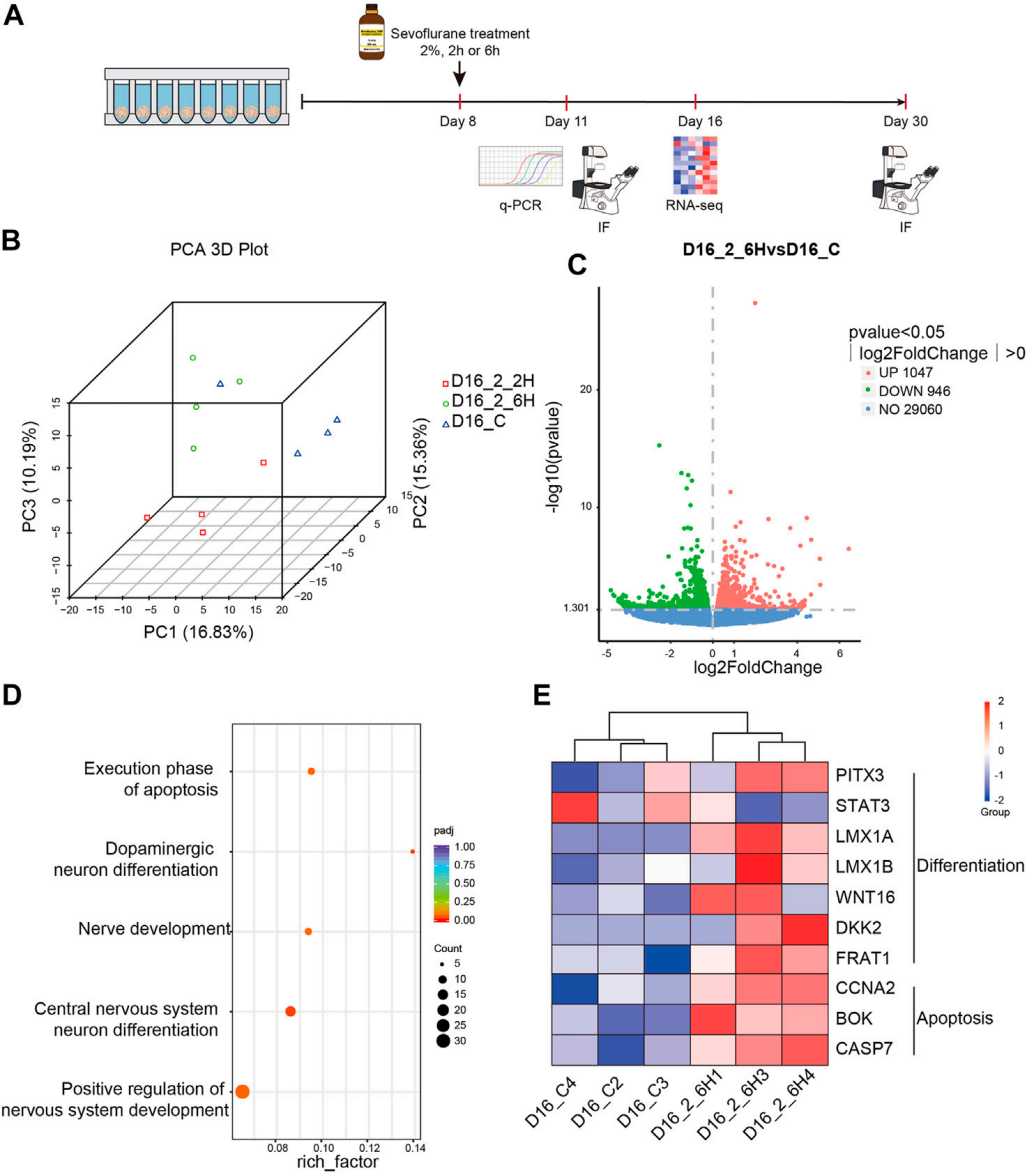
Sevoflurane exposure alters the transcription in hMBOs. **(A)** Principal Component Analysis (PCA) showed little difference within the group. **(B)** Volcanic map showed the differential gene expression between the MBO-SF-2%6H and control groups. **(C)** Gene Ontology (GO) of enriched genes in the MBO-SF-2%6H group compared with the control group. **(D)** Differential gene expression analysis showed the differential gene expression in neural differentiation and apoptosis between the MBO-SF-2%6H group and the control group. **(E)** Violin Plot showed the up-regulation of important transcription factors in the midbrain development of the MBO-SF-2%6H group compared with the control group.

To determine the effects of long-term sevoflurane exposure to hMBOs at the transcription level, transcriptome sequencing was performed on D16 (Figure 5A). PCA showed that sevoflurane exposure was responsible for most of the variance (PC1). In addition, a striking genotype effect was observed between the MBO-SF-2%2H and MBO-SF-2%6H groups (PCA3) (Figure 5B). We identified 1047 up-regulated and 946 down-regulated genes in the MBO-SF-2%6H group compared with the control group (Figure 5C). Subsequently, GO enrichment analysis of these DEGs in the MBO-SF-2%6H and control groups showed highly enriched categories related to the execution phase of apoptosis, dopaminergic neuron differentiation, nerve development and central nervous system neuron differentiation, and positive regulation of nervous system development (Figure 5D). Simultaneously, DEGs analysis showed that proliferation inhibitor genes, apoptosis-specific genes and neuronal differentiation-specific genes were up-regulated in the MBO-SF-2%6H group compared with the control group (Figure 5E). These results suggest that long-term exposure to sevoflurane could lead to premature differentiation of hMBOs.

### Long-term exposure to sevoflurane reduced the number of proliferating cells in hMBOs

**FIGURE 6:**
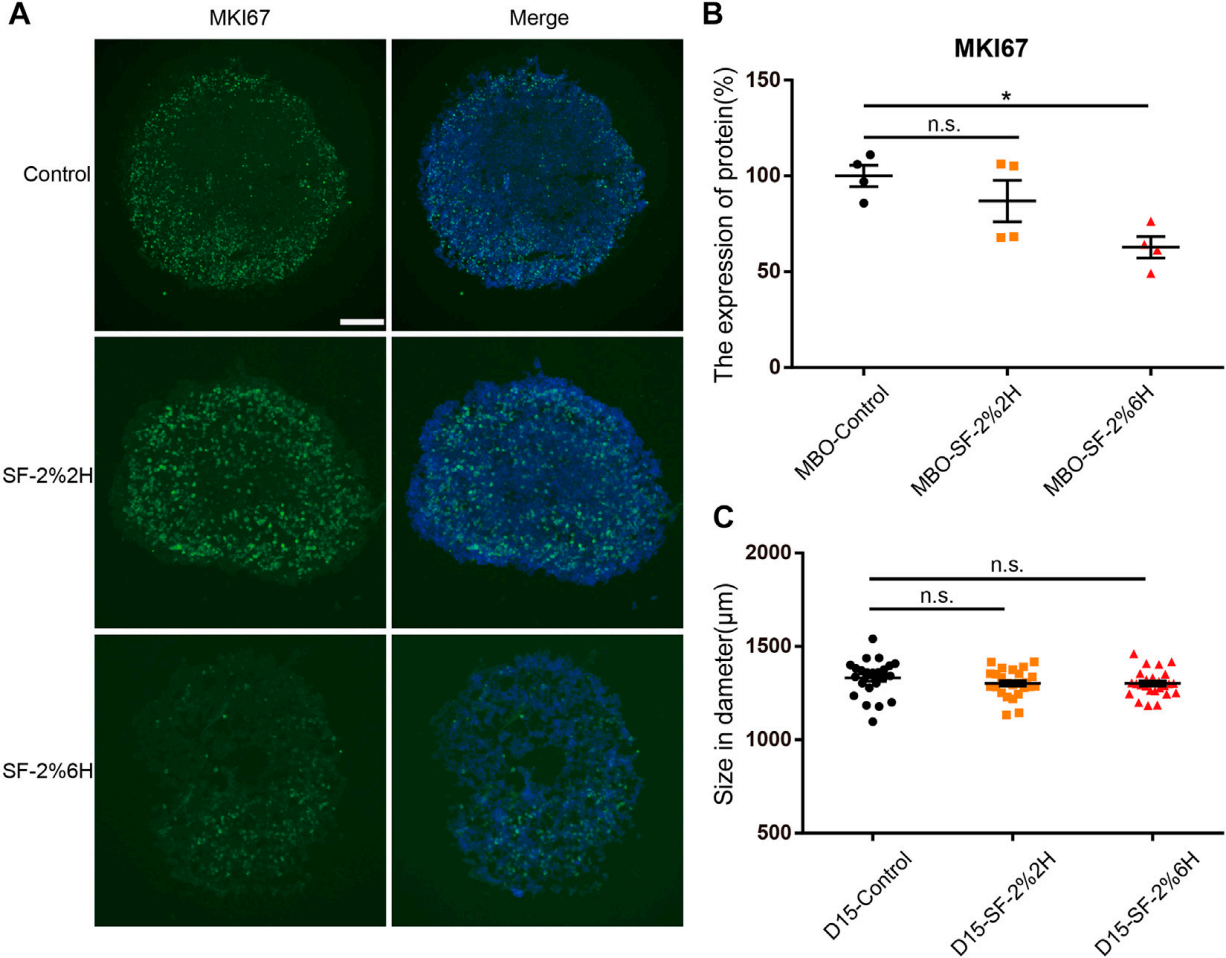
Sevoflurane decreased the cell proliferation number of hMBOs. **(A)** Immunofluorescence staining for MKI67 (green) with DAPI (blue) in hMBOs. **(B)** Quantification of **(A)** (n = 4). **(C)** Diameter of hMBOs (n = 25). Sample images of hMBOs slices were taken using an Olympus microscope IX83. Data represent mean ± SEM. Scale bars represent 200 μm.

Immunofluorescence for the proliferation marker Ki-67 (MKI67) was used to determine whether sevoflurane exposure could affect cell proliferation in hMBOs. The results showed that the number of MKI67-positive cells in the MBO-SF-2%2H group (86.9% ± 10.9%) was not significantly different from the control group (100% ± 5.5%). Conversely, the number of MKI67-positive cells in the MBO-SF-2%6H group (62.8% ± 5.6%) was significantly reduced compared with the control group (Figures 6A,B). The diameter of the hMBOs was not significantly different between the MBO-SF-2%6H group and the control group (Figure 6C). These results were similar to the results showing sevoflurane inhibiting the proliferation of neural stem cells in animal experiments and those of our monolayer dopaminergic neuron model.

### Long-term exposure to sevoflurane promotes premature of hDANs in hMBOs

**FIGURE 7:**
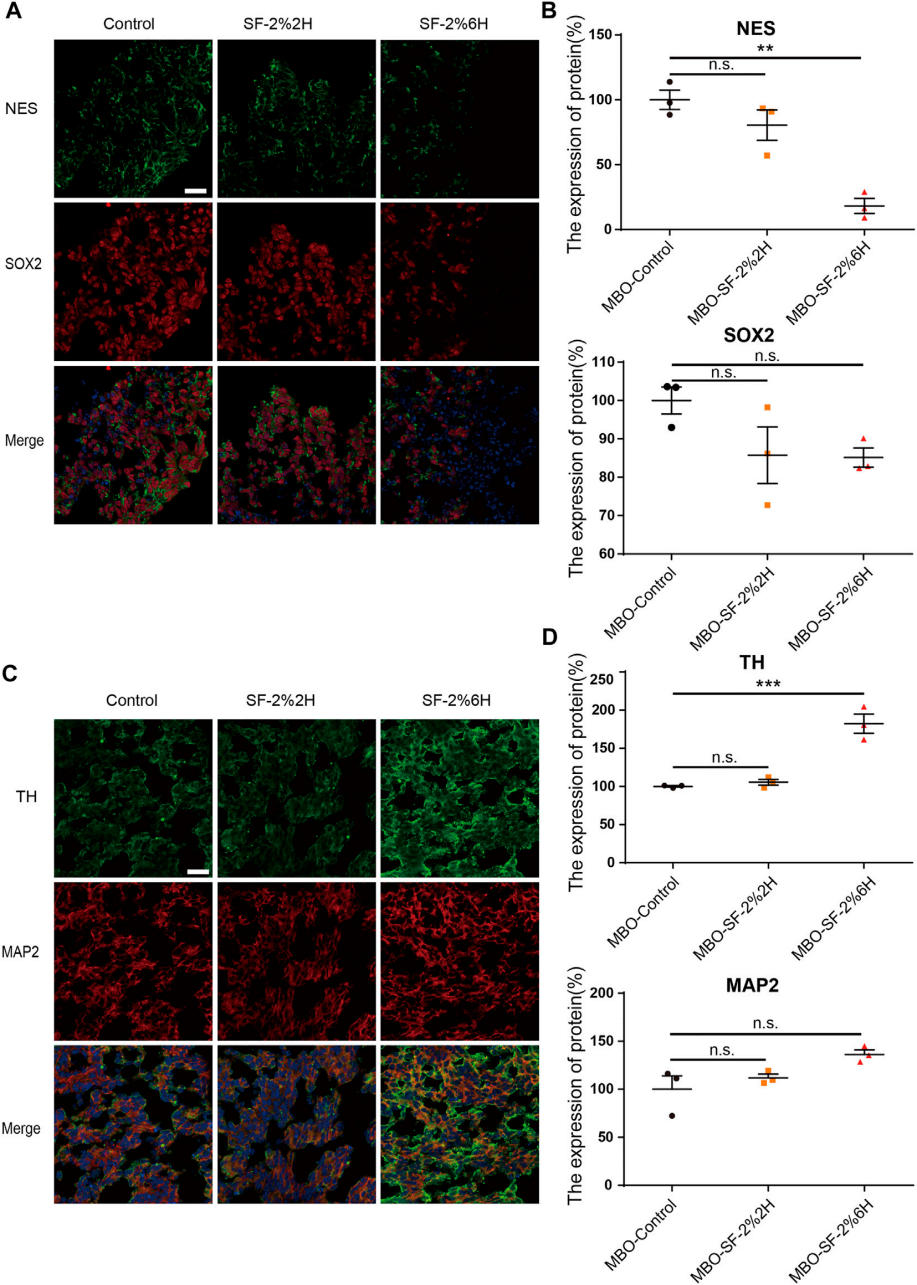
Sevoflurane promotes the early differentiation of hMBOs. **(A)** Sample images of hMBOs after 3 days of sevoflurane stimulation and immunofluorescence staining for NES (green), SOX2 (red) and DAPI (blue). **(B)** Quantification of NES (n = 3). **(C)** Quantification of SOX2 (n = 3). **(D)** Sample images of hMBOs after 20 days of sevoflurane stimulation and immunofluorescence staining for TH (green), MAP2 (red) and DAPI (blue). (E) Quantification of TH (n = 3). (F) Quantification of MAP2 (n = 3).

The immunofluorescence of stemness genes NES and SOX2 were used to identify neuron precursor cells. Our results showed a decreased NES expression in the MBO-SF-2%6H group compared with the MBO-control group (27.6% ± 0.2% VS. 100% ± 14.5%) (Figures 7A,B). The SOX2 expression has no significant change in the MBO-SF-2%6H group (85.1% ± 2.5%) nor the MBO-SF-2%2H group (85.7% ± 7.4%) compared with the MBO-control group (100% ± 3.5%). These results indicated that long-term exposure to sevoflurane decreased the number of neuron precursor cells. Then we used TH and MAP2 to identify hDANs, our results showed the expression of TH was significantly increased in the MBO-SF-2%6H group compared with the MBO-control group (246% ± 5.2% VS. 100% ± 28%) (Figures 7C,D). The expression of MAP2 has no significant change in the MBO-SF-2%6H group (136.3% ± 4.7%) nor MBO-SF-2%2H group (111.9% ± 3.8%) compared with the MBO-control group (100% ± 13.9%). These results indicated that long-term exposure to sevoflurane promoted the premature differentiation of dopamine neurons, but not all neurons.

Subsequently, we assessed the levels of several transcription factors, including NR4A2, LMX1A and ASCL1, in the differentiation of hDANs. Our results showed that the mRNA levels of NR4A2, LMX1A and ASCL1 were significantly up-regulated in the MBO-SF-2%6H group compared with the MBO-control group (Supplementary Figure S4), indicating that long-term exposure to sevoflurane promoted the premature differentiation of hDANs in hMBOs and that this effect could be realized by up-regulating the levels of transcription factors NR4A2, LMX1A and ASCL1.

### Mechanisms underlying long-term sevoflurane exposure facilitates the hMBO progenitor differentiation

**FIGURE 8:**
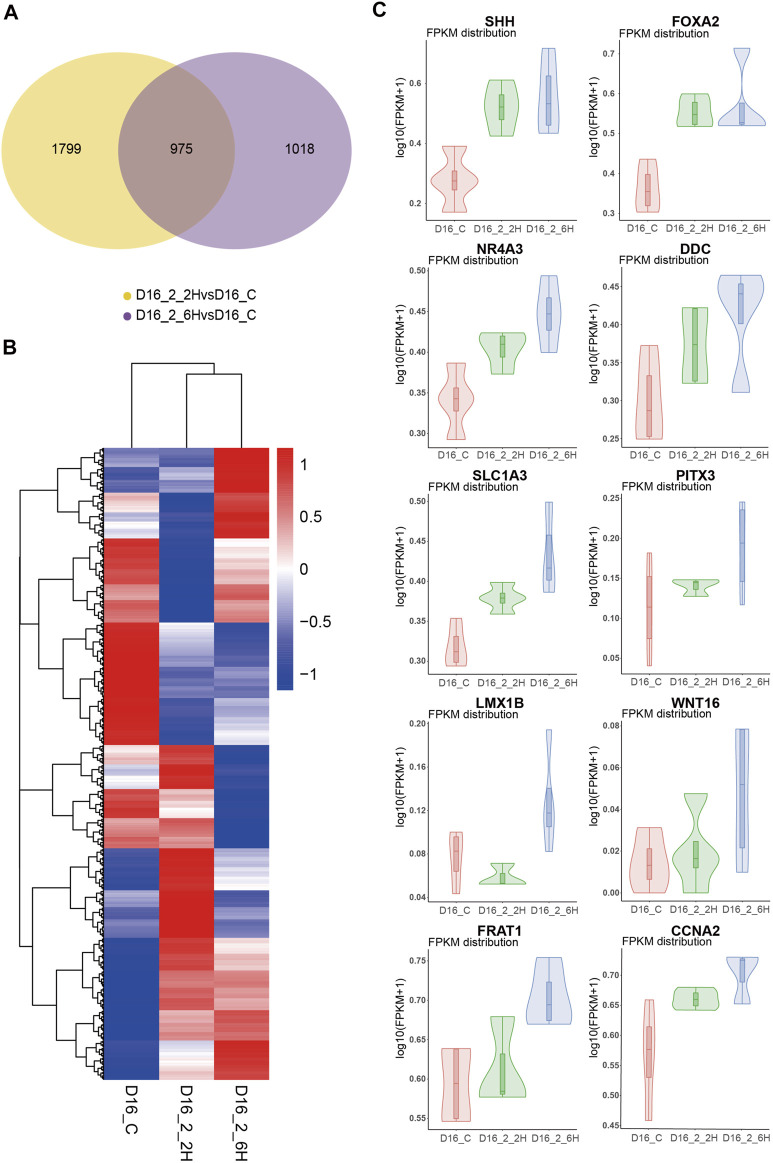
Mechanisms underlying sevoflurane exposure facilitates the hMBO progenitor differentiation. **(A)** Venn analysis showed the difference between the difference genes of MBO-SF-2%2H and control group and difference genes of MBO-SF-2%6H group and control group. **(B)** Volcanic map showed the differential gene expression between the MBO-SF-2%6H group, the MBO-SF-2%2H group and the control groups. **(C)** Violin Plot showed the up-regulation of important transcription factors in the midbrain development of the MBO-SF-2%6H group, MBO-SF-2%2H group compared with the control group.

To investigate the mechanisms underlying long-term sevoflurane exposure facilitates the hMBO progenitor differentiation, but not short-term sevoflurane exposure. Firstly, we performed differentially expressed gene (DEG) analysis, we analyzed the differences between the MBO-SF-2%2H group or the MBO-SF-2%6H group and the control group. We identified 2774 and 1993 DEG, respectively, from each of the above comparisons, with 975 genes at the intersection of the two (Figure 8A). Secondly, we analyzed the different gene expression patterns among the MBO-SF-2%6H group, the MBO-SF-2%2H group and the control group using all genes (Figure 8B), the volcanic map indicated the effects of short-term exposure to sevoflurane (MBO-2%2H) and long-term exposure to sevoflurane (MBO-2%6H) on the transcriptome of hMBOs have great differences. Finally, we focused on the expression of several key transcription factors during midbrain development. The Violin Plot showed the up-regulation of important transcription factors (SHH, FOXA2, NR4A3, DDC, SLC1A3, PITX3, LMX1B, WNT16, FRAT1, CCNA2) in the midbrain development of the MBO-SF-2%6H group, MBO-SF-2%2H group compared with the control group (Figure 8C). These results showed that short-term exposure to sevoflurane also up-regulated these transcription factors, however, the degree of up-regulation was far less than that of long-term exposure to sevoflurane.

## Discussion

Recently, there have been increasing clinical evidence and basic studies indicating that long-term sevoflurane exposure could induce multiscale neuropathological effects such ass cognitive impairment, neuronal apoptosis, synaptic deficiency and neuroinflammation. Due to limited access to human brain tissue, our understanding on the neuropathological changes caused by sevoflurane mainly depends on animal research models. However, these animal models failed to fully capture human-specific neuropathological features due to distinct differences between humans and mice in terms of the polarized neuroepithelium and intricate neurodevelopmental trajectory. Thus, there is a strong demand to develop more persuasive preclinical models for studying the causal relation between neuropathology and sevoflurane exposure.

DANs play a critical role in modulating the emergence of rats from sevoflurane anesthesia. A genetic lineage mapping study in mice recently reported that the midbrain FP selectively exhibited neurogenic potential and was the source of ventral midbrain DANs (Ono et al., 2007; Joksimovic et al., 2009; Fasano et al., 2010). hFPCs act as a critical signaling center during neural development along the ventral midline of embryos. However, there is no study has reported the effect of sevoflurane exposure on the differentiation of dopaminergic neurons, regardless of the biological model. Several previous studies focused on neuron pathology induced by sevoflurane but did not target the subtype of the neuron. Comparatively, our study focused on a subtype of the neurons (dopaminergic neurons), and the results showed that sevoflurane promoted premature differentiation of hDANs in hMBOs. The hMBO is an emerging hiPSCs-derived midbrain research model that closely emulates some critical neurophysiological features of the human midbrain including neurodevelopmental trajectory, neural cell diversity and cytoarchitectures. hMBO includes A9 and A10 subtype hDANs, astrocytes and oligodendrocytes (Monzel et al., 2017), it can more realistically mimic the human midbrain *in vitro*.

In this study, we generated human physiologically-related hMBOs, monolayer cultured hFPCs and hDANs derived from hiPSCs to investigate the influence of sevoflurane exposure on neuropathology associated with midbrain development. We found that 2% sevoflurane exposure for 6 h inhibited the proliferation of hFPCs, increased the apoptosis of hFPCs and promoted premature differentiation of hMBOs, while a short-time exposure to 2% sevoflurane had no significant effect on the proliferation and differentiation of hMBOs.

Several previous studies demonstrated that prenatal processes in inhaled anesthetics could lead to early-age brain growth defects such as abnormal cell proliferation, apoptosis and differentiation. Liu et al. reported that 6 h of stimulation with 4.1% sevoflurane was associated with cell cycle arrests in the G0/G1 phase, thus promoting the neuronal differentiation of primary mouse neural stem cells (Liu et al., 2018). However, using primary hippocampal neural stem cells from neonatal rats, Shao et al. found that 4.8% sevoflurane stimulation for 6 h inhibited hippocampal neural stem cell differentiation toward neurons (Shao and Xia, 2019). Comparatively, our results showed that long-term exposure to 2% sevoflurane promoted premature differentiation in hMBOs. The difference in these results could be due to the different mechanisms of sevoflurane in mice and human nervous systems or the different effects of sevoflurane on different types of neurons.

We also found that long-term exposure to sevoflurane led to proliferation inhibition and apoptosis promotion, which was consistent with some previous reports. For example, in a study by Zhang et al., the researchers exposed rats to 3% sevoflurane and found that repeated sevoflurane exposure acutely reduced neural progenitor proliferation (Zhang et al., 2015), and sevoflurane increased apoptosis in all brain regions (Lee et al., 2017; Perez-Zoghbi et al., 2017; Ikonomidou et al., 2019). Specifically, our analysis focuses on hFPCs and hDANs, which demonstrated that long-term sevoflurane exposure could have extensive side effects on fetal brain development. One of the key findings from our study was that long-term exposure to sevoflurane was associated with dysregulated neurogenesis in human fetal midbrain development.

Further, we speculated that the premature differentiation of hFPCs caused by sevoflurane could reduce the number of hFPCs, long-term exposure to sevoflurane would reduce the proliferation and promote apoptosis of hFPCs, and sevoflurane could lead to long-term reduction of dopaminergic neurons. This may be because long-term exposure to sevoflurane up regulates some transcription factors in the differentiation of dopaminergic neurons, while short-term exposure to sevoflurane has a weak up-regulation effect on these transcription factors.

Remarkably, these cell fate changes seen in organoids were distinct from the cell fate observed in monolayer neural cultures (Cairns et al., 2020). These discrepancies were likely due to major changes in cell adhesion and polarity of neural cells grown in monolayer and three-dimensional organoid cultures (Scuderi et al., 2021), confirming the superiority of brain organoids in mimicking the neuropathological features associated with sevoflurane exposure. The differences between the monolayer culture system and the organoid culture system in developmental biology, disease modeling and drug screening have been increasingly observed. Veronica et al. demonstrated that the type I interferon could reverse the nerve defects caused by the Zika virus and herpes simplex virus one in their organoid model, although type I interferon failed to reverse these defects in the monolayer neural culture models (Krenn et al., 2021). Recently, cerebral organoids have been used in sevoflurane studies, and it was found that sevoflurane exposure impaired the nuclear migration in cerebral organoids (Jiang et al., 2021). Thus, hMBO provides a highly relevant *in vitro* human model for studying the persistent effects of sevoflurane on dopaminergic neuronal development.

This study had several limitations. First, we examined only one anesthetic agent at a single concentration, which restricts the generalizability of our results, but the condition closely resembled clinical settings in terms of dosage and duration of exposure. Second, the targets through which sevoflurane caused premature differentiation of hDANs should be further investigated in follow-up studies.

In summary, this study revealed that long-term exposure to sevoflurane inhibited hFPCs proliferation, promoted hFPCs apoptosis and facilitated hFPCs-directed differentiation toward hDANs. The results imply that clinical application of sevoflurane for long duration or repeated anesthesia may lead to disorders during fetal midbrain development. Our study opens a new avenue for studying the neuropathological features of sevoflurane-stimulated human fetal brain development *in vitro* and provides new insights into the neurotoxic effects of sevoflurane on a specific fetal brain region by establishing a high-fidelity *in vitro* model.

## Conclusion

This study revealed that long-term exposure to sevoflurane could promote the premature differentiation of hMBOs, while short-term exposure had negligible effects, suggesting that long-term exposure to sevoflurane in pregnant women may lead to fetals’ midbrain development disorder.

## Data Availability

The datasets presented in this study can be found in online repositories. The names of the repository/repositories and accession number(s) can be found in the article/supplementary material.
