# Supplementary material for: Sevoflurane promotes premature differentiation of dopaminergic neurons in hiPSC-derived midbrain organoids
Source: Front Cell Dev Biol. 2022 Sep 13;10:941984. doi: 10.3389/fcell.2022.941984 (PMC9513420; doi:10.3389/fcell.2022.941984)
Supplement: Supplementary file 1 [file DataSheet1.PDF]

Supplementary Material

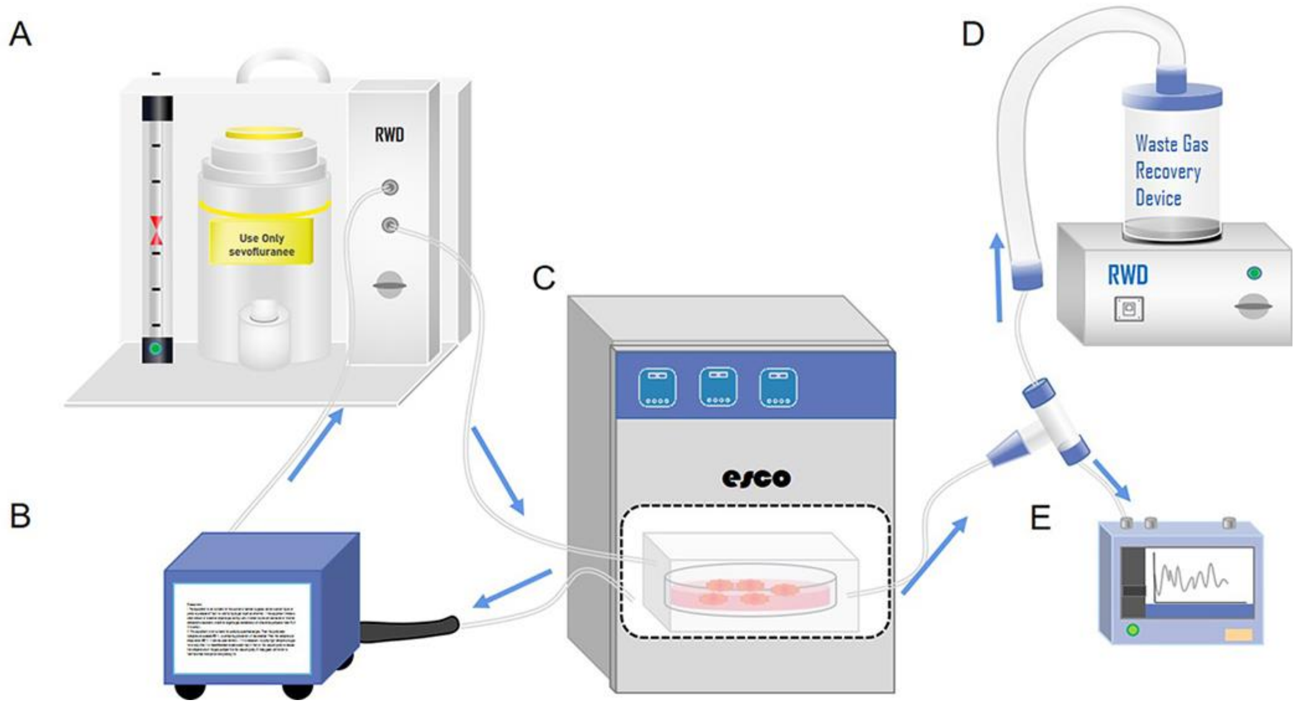

Supplementary Figure 1. Sevoflurane exposure device. (A) sevoflurane volatile tank (B)a pump (C) an exposure box, (E)a sevoflurane sensor (D)a waste gas recovery device.

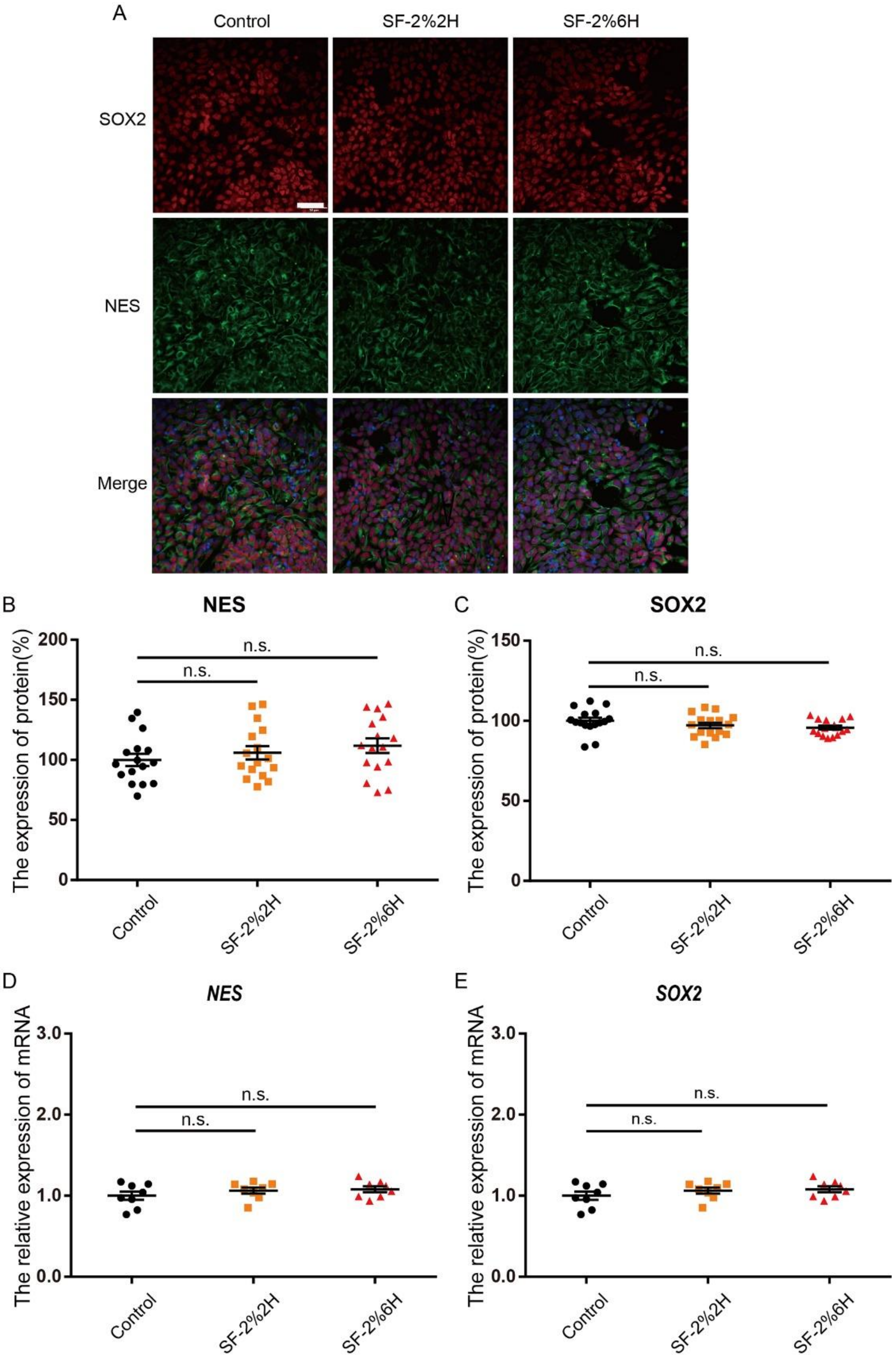

Supplementary Figure 2. Sevoflurane had no significant effect on the differentiation of hFPCs. (A) Sample images of hFPCs after sevoflurane stimulate 3 days under proliferation condition, immunofluorescence staining for NES (green)、SOX2 (red) and DAPI (blue). (B) Quantification of NES and SOX2(n = 16). (C) Detection of the mRNA expression of SOX2 and NES (n = 8). The images were taken using an Olympus microscope BX53. Data represent the mean ± SEM.

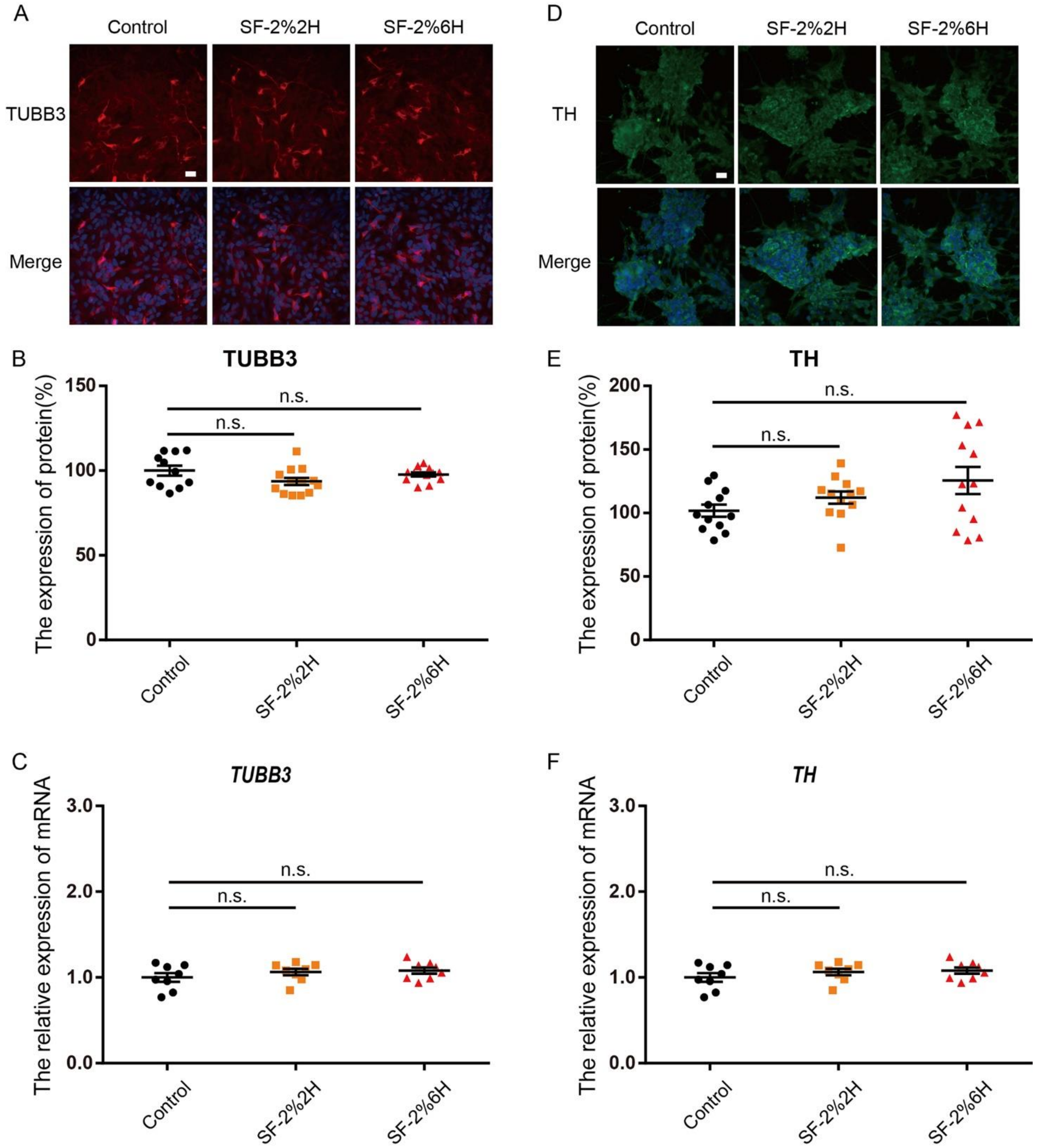

Supplementary Figure 3. Sevoflurane had no significant effect on the differentiation of hFPCs - derived monolayer hDANs. (A) Sample images of hFPCs after sevoflurane stimulate 3 days under differentiation condition, immunofluorescence staining for TUBB3 (red) and DAPI (blue). (B) Quantification of TUBB3(n = 12). (C) Detection of the mRNA expression of TUBB3(n = 8). (D) Sample images of hDANs after sevoflurane stimulation, immunofluorescence staining for TH (green) and DAPI (blue). (E) Quantification of TH (n = 12). (F) Detection of the mRNA expression of TH (n = 8). The images were taken using an Olympus microscope BX53. and the quantifications using Image J. Data represent the mean ± SEM.

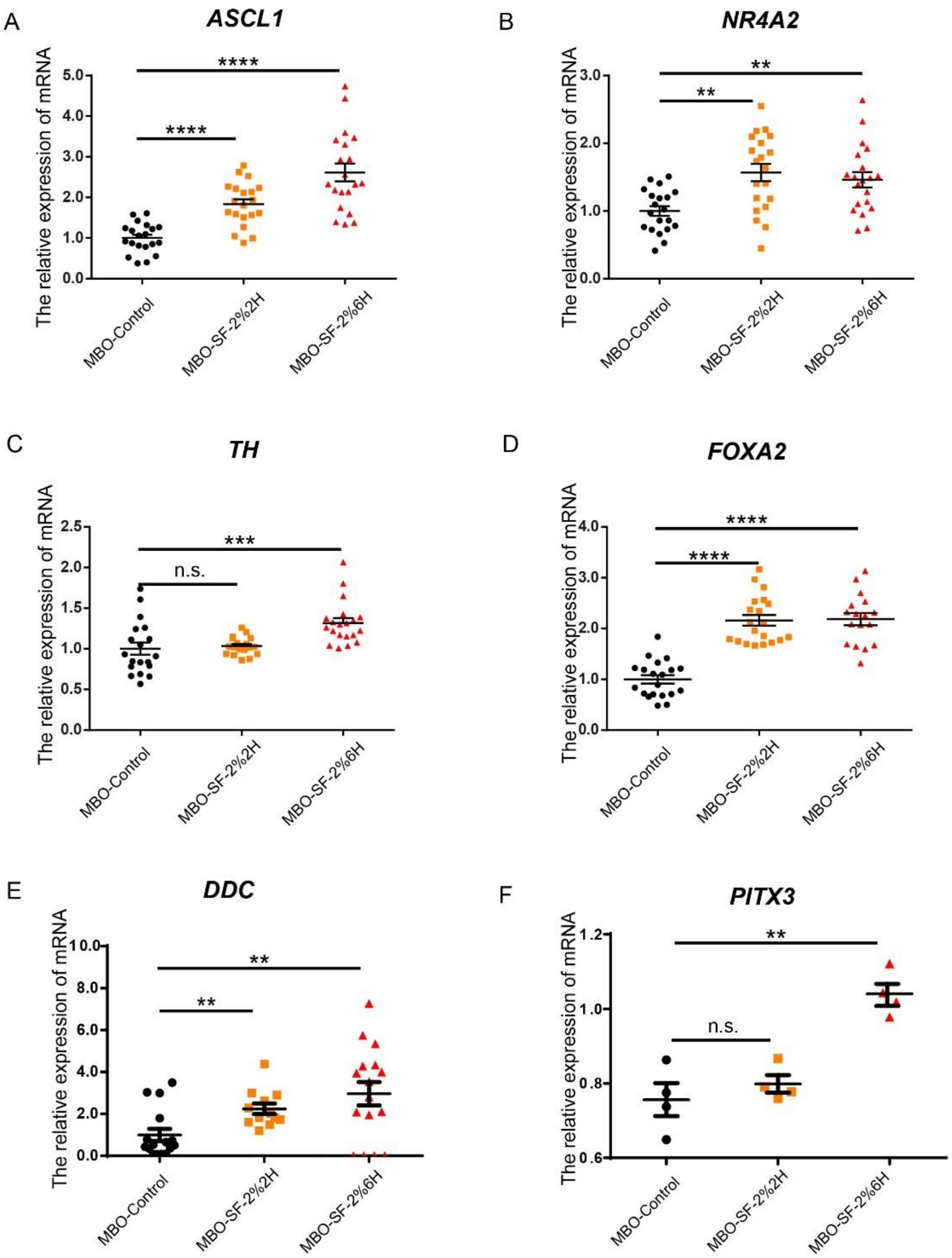

Supplementary Figure 4. Sevoflurane exposure promotes premature differentiation of dopaminergic neurons at the mRNA level. (A-F) Detection of the mRNA expression of ASCL1, NR4A2, TH, FOXA2, DDC and PITX3.

Supplementary Table 1. N2B27 medium

Component	Company	Cat. No	Volume or concentration
**DMEM-F12**	Invitrogen	10-565-018	50 mL
**Neurobasal**	Invitrogen	21103-049	50 mL
**N2 supplement**	Invitrogen	17504-044	1:200
**B27 supplement lacking Vitamin A**	Invitrogen	12587010	1:100
**L-glutamine**	Invitrogen	35050-061	1:100
**penicillin/streptomycin**	Invitrogen	15140163	1:100

Supplementary Table 2. N2B27 maintenance medium

Component	Company	Cat. No	Concentration
**CHIR-99021**	Selleck	S2924	3 μM
**purmorphamine**	StemCell Technologies	72202	0.75 μM
**ascorbic acid**	Sigma	A5960	150 μM

Supplementary Table 3. N2B27 differentiation medium

Component	Company	Cat. No	Concentration
**hBDNF**	Peprotech	450-02	10 ng mL^{-1}
**hGDNF**	Peprotech	450-10	10 ng mL^{-1}
**dbcAMP**	Sigma	D0627	500 μM
**ascorbic acid**	Sigma	A5960	200 μM
**TGF-β3**	StemCell Technologies	78131	1 ng mL^{-1}
**purmorphamine**	StemCell Technologies	72202	1 μM

Supplementary Table 4. Antibodies

Antibody	Host species	Company	Cat. No	Dilution or concentration
**Nanog**	Goat	R&D	AF-1997	5 μg mL^{-1}
**Oct3/4**	Mouse	Santa Cruz	SC-5279	1:200
**OTX2**	Goat	R&D	AF-1979	10 μg mL^{-1}
**SOX2**	Rabbit	Boster	BA3292	1:200
**NES**	Mouse	Invitrogen	14984382	1:200
**TUBB3**	Rabbit	CST	5568	1:200
**MAP2**	Rabbit	CST	4542	1:200
**TH**	Mouse	R&D	MAB7566	10 μg mL^{-1}
**ASCL1**	Mouse	R&D	MAB2567	10 μg mL^{-1}
**MKI67**	Mouse	CST	9449	1:500
**Alexa Fluor 568**	Donkey anti-Rabbit	Invitrogen	A10042	1:500
**Alexa Fluor 488**	Donkey anti-Mouse	Invitrogen	A21202	1:500
**Alexa Fluor 568**	Donkey anti-Goat	Invitrogen	A11057	1:500

Supplementary Table 5. Primers

Gene	Forward	Reverse
GAPDH	GGACCTGACCTGCCGTCTAG	GTAGCCCAGGATGCCCTTGA
Sox2	CATCCACACTCACGCAAA	CTCCCCAGGTTTTCTCTGT
NES	GTGAAGGGAGGGGCTGA	ACCAGGGGCTCTATCGC
TUBB3	AGACGCGCCCAGTATGA	GTCGCCCACGTAGTTGC
TH	GGGCTGTGTAAGCAGAACG	AAGGCCCGAATCTCAGGCT
FoxA2	GGGGTAGTGCATCACCTGTT	CCGTTCTCCATCAACAACCT
NR4A2	GCACTCCGGGTCGGTTTAC	GCCACGTAGTTCTGGTGGAA

ASCL1	CTTGTCAGTGGCGTTGG	TAGTTGGCGATGGGGTT
EN1	TCTCGCTGTCTCTCCCTCTC	CGTGGCTTACTCCCCATTTA
MAP2	CCGAGTGAGAAGAAGGTC	TCTGGCAGTGGTTGGTTA
